# IL-16 and BCA-1 Serum Levels Are Associated with Disease Severity of *C. difficile* Infection

**DOI:** 10.3390/pathogens10050631

**Published:** 2021-05-20

**Authors:** Dor Gotshal, Maya Azrad, Zohar Hamo, Orna Nitzan, Avi Peretz

**Affiliations:** 1 The Azrieli Faculty of Medicine, Bar-Ilan University, Safed 1311502, Israel; dor.gotshal@gmail.com (D.G.); Zhamo@poria.health.gov.il (Z.H.); ONitzan@poria.health.gov.il (O.N.); 2 Clinical Microbiology Laboratory, The Baruch Padeh Medical Center, Poriya, Tiberias 1528001, Israel; mazrad@poria.health.gov.il; 3 Unit of Infectious Diseases, The Baruch Padeh Medical Center, Poriya, Tiberias 1528001, Israel

**Keywords:** *Clostridioides difficile*, nosocomial infection, cytokines, chemokines, disease severity

## Abstract

*Clostridioides difficile* infection (CDI) is associated with a high risk for complications and death, which requires identifying severe patients and treating them accordingly. We examined the serum level of six cytokines and chemokines (IL-16, IL-21, IL-23, IL-33, BCA-1, TRAIL) and investigated the association between them and patients’ disease severity. Concentrations of six cytokines and chemokines were measured using the MILLIPLEX^®^MAP kit (Billerica, MA, USA) in serum samples attained from CDI patients within 24–48 h after laboratory confirmation of *C. difficile* presence. Demographic and clinical data were collected from medical records. The disease severity score was determined according to guidelines of the “Society for Healthcare Epidemiology of America and the Infectious Diseases Society of America” (SHEA-IDSA). Out of 54 patients, 20 (37%) had mild to moderate disease and 34 (63%) had severe disease. IL-16 (*p* = 0.005) and BCA-1 (*p* = 0.012) were associated with a more severe disease. In conclusion, IL-16 and BCA-1, along with other cytokines and chemokines, may serve as biomarkers for the early prediction of CDI severity in the future. An improved and more accessible assessment of CDI severity will contribute to the adjustment of the medical treatment, which will lead to a better patient outcome.

## 1. Introduction

In the last two decades, *C. difficile* infection (CDI) has become the most common nosocomial infection acquired in hospitals and institutions for prolonged hospitalization, especially in developing countries [1].

Recent antibiotic use is one of the main risk factors that contribute to CDI development [2]. Antibiotics cause a disruption of the intestinal microbiota that allows the germination of bacterial spores followed by the proliferation of the vegetative cells. Other risk factors are an age ≥ 65 years, long-term stays in healthcare facilities, recent gastrointestinal surgery, immunocompromised conditions, use of antineoplastic agents, gastric acid suppressants, and comorbidities such as inflammatory bowel disease (IBD) [2,3,4,5,6,7,8,9,10,11,12].

The pathogenesis of CDI depends on several virulence factors that contribute to disease development and severity; the main cause is the production of two toxins, toxin A and toxin B with glycosyltransferase activity. Rho proteins, the toxins’ target, are located in the cytosol and are involved in numerous signal processes including actin cytoskeleton regulation, cell cycle progression, and gene transcription, as well as the control of kinases activity [13]. In addition, these toxins cause an increase in intestinal permeability and fluid accumulation that leads to diarrhea, which is one of the hallmarks of CDI [14].

While the bacterial contribution to the disease hallmark is well studied, less is known regarding the effect of the host immune response on disease development and complications.

During the appearance of bacterial infections, such as toxin producing *C. difficile*, the innate immune system is activated, primarily in the intestinal mucus. This reaction leads to the secretion of a variety of proinflammatory cytokines, which are used as messengers that transmit messages between different cells, including cells of the immune system. This leads to the secretion of other cytokines that propagate the inflammatory process.

Several studies have shown the involvement of cytokines in CDI. For example, the cytokine IL-8 activates the chemotaxis mechanism for neutrophils recruitment and the activation of innate lymphoid cells [15]. Previous studies have shown that fecal IL-8 levels were high in samples of CDI patients and were positively correlated with disease severity [16,17]. Another work has reported that the concentration ratios of IL-1β/IL-receptor a (IL-ra) were significantly increased in patients with severe CDI compared to patients with mild CDI [16]. Similar to IL-8, IL-1β is essential for host responses and provides protection following infection and injury [18]. It is produced and released by a wide variety of cells such as intestinal epithelial cells, dendritic cells, and macrophages [19].

All these studies suggest that cytokines may serve as a specific biomarker that can predict disease severity. Currently, there is no reliable marker that assesses CDI severity or predicts treatment failure. Additionally, treatment is often similar for both moderate and severe cases [20]. Due to the high risk of complications and high mortality rate among CDI patients, it is valuable to find a biomarker that can be easily and rapidly detected at the disease onset, in order to predict the disease severity and, accordingly, better adjust the treatment.

We have previously described the association of several cytokines and the disease severity of CDI (Hamo et al., 2019). Here, we investigated an additional six cytokines that were not yet extensively studied in relation to CDI—IL-16, IL-21, IL-23, IL-33, BCA-1, and TRAIL—and investigated whether their serum levels were associated with the disease severity.

## 2. Results

The study included fifty-four patients aged 46–98 years (mean age, 76.6 years, 61.1% female) that were positive for *C. difficile*. Table 1 summarizes the clinical and demographic characteristics of the patients. The majority of CDI cases (37/54, 68.5%) were nosocomial, while the rest were acquired in the community. CDI severity was mild to moderate in 20 patients (37%) and severe in 34 patients (63%). In most patients (34/54, 63%), both toxins A + B were found. Six (11.1%) stool samples contained only toxin A and 14 (25.9%) contained only toxin B.

### Characterization of the Immune Response in CDI Patients

**Figure 1:**
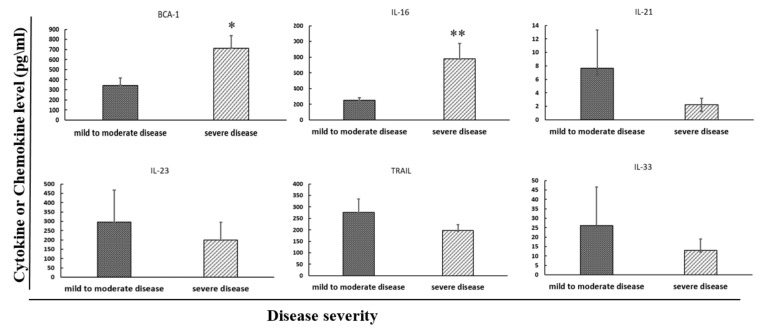
Serum cytokine levels correlated with disease severity of CDI patients. In BCA-1 and IL-16, severe CDI patients had higher cytokines levels than mild patients. * *p* < 0.05, ** *p* < 0.01.

With the intention of finding a biological marker that could correlate with disease severity, the concentrations of six cytokines and chemokines were measured in patients’ serum and compared between patients with mild to moderate and severe CDI (Table 1). Out of these six serum cytokines, significantly higher levels from two cytokines, IL-16 (*p* = 0.005) and BCA-1 (*p* = 0.012), were associated with a more severe disease using the “Society for Healthcare Epidemiology of America and the Infectious Diseases Society of America” (SHEA-IDSA) criteria. (Table 2, Figure 1).

The study does not show an association between the cytokines and the gender, the type of toxin produced by the bacteria, the in-hospital mortality, and infection acquisition (Table 3). Additionally, there was no association between the cytokines levels and the patients’ age (*p* > 0.05 for all markers).

Next, we investigated the association of the six cytokines’ levels with other cytokines (Table 4). BCA-1 levels were positively correlated with creatinine levels (*p* < 0.05) and lymphocytes’ levels (*p* < 0.001) and were negatively correlated with neutrophils levels (*p* < 0.01). IL-16 levels were positively correlated with the white blood cell count (*p* < 0.05). TRAIL levels were positively correlated with albumin levels (*p* < 0.001) and negatively correlated with creatinine levels (*p* < 0.05) and CRP levels (*p* < 0.05). However, most of the correlation coefficients were distant from the value 1, indicating weak associations.

## 3. Discussion

In this study, we investigated the associations of the serum levels of six cytokines and chemokines—IL-16, IL-21, IL-23, IL-33, BCA-1, and TRAIL—with CDI severity.

Our findings identified an association between the levels of two cytokines and the disease severity: IL-16 and BCA-1. These results are consistent with the results of others who have shown that *C. difficile* stimulates the production of multiple cytokines and chemokines, which is considered a characteristic of CDI.

IL-16 is a pleiotropic cytokine that chemoattracts active T cells. This cytokine signaling process is mediated by CD4-positive cells. IL-16 is released by various cells including lymphocytes and epithelial cells. Originally, this interleukin was thought to be an attractor of active T-cells [21]. Later, studies have shown that IL-16 plays a role in the attraction and activation of a variety of other cells that express the CD4 molecule. These cells include eosinophils, monocytes, and dendritic cells [22]. Additionally, IL-16 was associated with the stimulation of proinflammatory cytokines’ production, including Il-6, tumor necrosis factor alpha (TNFα), IL-1α, and IL-15 [23,24]. Therefore, it is possible that higher levels of IL-16 contribute to higher levels of other cytokines and the activation of various immune cells, resulting in a more profound inflammatory response to *C. difficile*.

We demonstrated a statistical significance between the serum IL-16 level and patients’ severity of disease, suggesting that IL-16 and T cells contribute to immunopathology and to the course of *C. difficile*-mediated disease. These findings suit other studies that showed a positive association between IL-16 and the severity of CDI [25] and studies that reported an association of IL-16 with other intestinal diseases including Crohn’s disease and ulcerative colitis [24]. B cell-attracting chemokine 1 (BCA-1), also known as CXCL-13, is a chemokine that is selectively chemotactic for B cells, both B-1 and B-2 subsets. This chemokine achieves its effect by interacting with the chemokine receptor CXCR5. Both BCA-1 and CXCR5 were expressed in high numbers in the guts, lymph nodes, spleen, and the liver [26,27].

We demonstrated a statistical significance between the serum BCA-1 level and the patients’ severity score for disease. These findings suggest that BCA-1 also has a major role in contributing to the immunopathology and the course of *C. difficile*-mediated diseases. As BCA-1 attracts B cells, it is possible that higher levels of this chemokine induce the activation of a large amount of B cells, resulting in a more aggressive inflammatory response. A future study should test whether severe CDI patients have higher levels of antibodies against bacterial toxins when compared to mild patients, as antibodies are produced by C cells. To the best of our knowledge, our study is the first that shows that serum BCA-1 levels are associated with CDI severity. Previous studies have shown an association of BCA-1 levels with disease severity in infected children with *helicobacter pylori* and in adults with gastric cancer [28]. Other studies reported a poor prognosis in a few types of cancer including gastric cancer and prostate cancer [29,30].

We expected to find increased levels of IL-21 and IL-23 in more severe CDI, since both have a proinflammatory effect. Furthermore, a previous study in mice has shown an association between *C. difficile* toxin B and the excretion of IL-21 by iNKT [31]. Another study has shown that CDI-infected mice that were unable to produce IL-23 did not die [32]. However, no statistically significant differences were noted in the level of these cytokines between mild to moderate and severe CDI. A possible explanation is that the patients’ comorbidities masked part of the immune response.

The study has several limitations. First, the study population was small. Second, although the serum sampling was done within 24–48 h of receiving a positive result for *C. difficile* presence, the samples may not have been collected at the same stage in terms of the immune response. We suggest that future studies should collect several blood samples on sequential days during hospitalization to detect the time point at which a change in cytokine levels occurs. Finally, our study was performed on patients positive to *C. difficile,* and no comparison was made to other intestinal diseases.

## 4. Materials and Methods

### 4.1. Study Population and Sample Collection

The study population consists of patients above the age of 18 years who were diagnosed with CDI at the Poriya Baruch Padeh Medical Center between the years 2015–2019. CDI was confirmed by stool examination using the GeneXpert toxigenic *C. difficile* polymerase chain reaction (PCR) assay (Cepheid, Sunnyvale, CA, USA), identifying three targets: Toxin B, Binary Toxin, and the presence of *tcd*C deletion.

Patients had to sign a consent form or had a legal guardian sign in their place. Pregnant women, patients with pneumonia, patients with sepsis due to causes other than CDI, patients with bacteremia, and patients suffering from mental illness were excluded from this study.

This study received the approval of the Institutional Committee of Helsinki of the Medical Center, approval No. POR-0085-15.

Serum and stool samples were collected from each patient.

### 4.2. Measurement of Cytokine Concentrations–IL16, IL-21, IL-23, IL-33, BCA-1, TRAIL

Cytokine and chemokine concentrations were measured using the MILLIPLEX^®^MAP kit (Billerica, MA, USA) based on the Luminex xMAP^®^ technology. This technology is based on fluorescent-coded magnetic beads that are coated with a specific capture antibody for each cytokine. First, a 25 μL serum was added into each well in a 96-wells plate. Then, 25-μL magnetic beads coated with specific monoclonal antibodies were introduced to each well. After an overnight incubation and washing steps, 25 μL of Detection Antibodies was added to each well. These are secondary antibodies chemically conjugated to Biotin. After a one-hour incubation, 25 μL of streptavidin-phycoerythrin was added. After a short incubation and washing steps, the fluorescence of the plate was red when using the Luminex 200™ reader. Each cytokine has a different color.

### 4.3. Disease Severity Scoring and Demographic Data Collection

The *C. difficile* severity score index was calculated using a severity score index according to the guidelines of the “Society for Healthcare Epidemiology of America and the Infectious Diseases Society of America” (SHEA-IDSA). To this end, the following demographic data were collected from the medical records: age, gender, functional status, community versus nosocomial acquired CDI, and death during hospitalization. According to this score, CDI patients were divided into groups of mild to moderate disease and severe disease: mild to moderate disease: ¼ leukocytosis ≤ 15,000 cells/mL and creatinine ≤ 1.5 times the premorbid level; severe disease: ¼ leukocytosis ≥ 15,000 cells/mL or serum creatinine ≥ 1.5 times the premorbid level. Severe disease was also defined as a serum albumin level of ≤3 g/dL at the time of active infection [33].

### 4.4. Toxin Detection

Toxins A and B were detected using the CerTest *Clostridioides difficile* GDH+ Toxin A+B kit according to the manufacturer’s instructions (Certest Biotec, S.L, San Mateo de Gállego, Zaragoza, Spain). The kit is a colored chromatographic assay for the qualitative detection of *C. difficile* antigen glutamate dehydrogenase (GDH) and Toxins A and B in stool samples. The sample was mixed with a test solution that contained mouse monoclonal antibodies anti-GDH/Toxin A/Toxin B conjugated to red polystyrene latex. When GDH antigen/Toxin A/Toxin B is present in the sample, the antigen/toxin reacts with its specific antibodies in the test solution, and this complex is captured by the antibodies in the test strips, resulting in a visible red line.

### 4.5. Statistical Analysis

Chi-square was applied to analyze the differences in distribution of the categorical parameters between mild and moderate disease severity. The nonparametric Wilcoxon–Mann–Whitney Rank sum test or Analysis of Variance (ANOVA) was applied to analyze the differences in the continuous parameters between mild and moderate disease severity. A Pearson correlation was performed to analyze the associations between cytokines levels and other serum inflammatory markers’ levels. All tests were applied as two-tailed, and a p value of 5% or less was considered statistically significant. The data was analyzed using SAS^®^ version 9.3 (SAS Institute, Cary, NC, USA).

## 5. Conclusions

Our results point to the involvement of two proinflammatory cytokines in CDI pathogenesis, which are involved in the recruitment and activation of various cells from humans’ immunity system. As their levels are correlated with disease severity, further investigations should be performed to understand the specific contribution of these cytokines to CDI pathogenesis. Furthermore, these cytokines may serve as effective diagnostic tools to predict disease severity. The measurement of these cytokines is easy and quick. Consequently, we believe that in the future, testing serum cytokines levels may be introduced into the routine blood examinations of CDI patients; this may be effective in predicting the disease severity.

An improved and more accessible assessment of CDI severity will contribute to an adjustment of the medical treatment, which will lead to better patient care and will hopefully reduce the patient’s mortality. Further prospective studies need to be conducted in order to investigate the efficacy of proinflammatory cytokines in the diagnosis and later phases of CDI, as an alternative or as a supplement to today’s procedures.

## Figures and Tables

**Table 1:** Clinical and demographic characteristics of patients with *Clostridioides difficile* infection (CDI).

Parameter	*n* (%)
**Gender**	
**Male**	21 (38.9)
**Female**	33 (61.1)
**Disease severity**	
**Mild to moderate**	20 (37)
**Severe**	34 (63)
**Toxins Presence**	
**Toxin A**	6 (11.1)
**Toxin B**	14 (25.9)
**Toxins A + B**	34 (63)
**In-hospital mortality**	
**Alive**	39 (72.2)
**Died**	15 (27.8)
**Infection acquisition**	
**Nosocomial**	37 (68.5)
**Community**	17 (31.5)

**Table 2:** Serum cytokine levels in patients with CDI, by disease severity *.

	Mild-Moderate (*n* = 20) Mean (Range)	Severe (*n* = 34) Mean (Range)	*p*-Value
**IL-16**	250.3 (0–622.1)	778 (0–6578.3)	0.005 **
**IL-21**	7.7 (0–110.9)	2.2 (0–27.7)	0.956
**IL-23**	294.8 (0–3083.6)	198.9 (0–2638.1)	0.740
**IL-33**	26 (0–408.8)	12.9 (0–174.1)	0.758
**BCA-1**	344.7 (63.2–1000)	712.8 (39.6–4266)	0.012 **
**TRAIL**	276 (16.9–881.1)	190.8 (0–584.3)	0.333

* As determined using SHEA-IDSA criteria. ** Statistically significant.

**Table 3:** Serum cytokine levels in CDI patients by patient characteristics.

	*p*-Value					
Parameter	IL-16	IL-21	IL-23	IL-33	BCA-1	TRAIL
**Gender (Male/Female)**	0.531	0.182	0.360	0.751	0.487	0.401
**Toxins Presence (Toxin A/B/A + B)**	0.215	0.391	0.851	0.936	0.543	0.098
**In-Hospital Mortality (Alive/Dead)**	0.284	0.792	0.227	0.207	0.309	0.380
**Infection Acquisition (Nosocomial/Community)**	0.635	0.474	1	0.600	0.322	0.402

**Table 4:** Correlations between serum cytokine levels and other inflammatory markers.

	Pearson Coefficient					
Parameter	IL-16	IL-21	IL-23	IL-33	BCA-1	TRAIL
**Creathinine**	−0.06	−0.04	−0.05	0.06	0.31 *	−0.35 *
**Albumin**	0.06	−0.07	−0.21	0.02	−0.11	0.46 ***
**CRP**	−0.15	−0.05	−0.07	0	0.06	−0.28 *
**WBC**	0.30 *	−0.09	−0.05	0	−0.06	−0.10
**Neutrophils**	0.15	0.07	0.07	0.09	−0.42 **	−0.11
**Lymphocytes**	−0.13	−0.08	−0.03	−0.10	0.45 ***	0.06
**Calprotectin**	0.01	0.02	0.12	0.16	−0.04	0.13
**Procalcitonin**	0.03	−0.05	−0.05	−0.02	0.02	−0.16

* *p* < 0.05, ** *p* < 0.01, *** *p* < 0.001.

## Data Availability

The datasets used and/or analyzed during the current study are available from the corresponding author on reasonable request.
